# Simulation of Postsynaptic Glutamate Receptors Reveals Critical Features of Glutamatergic Transmission

**DOI:** 10.1371/journal.pone.0028380

**Published:** 2011-12-15

**Authors:** Renaud Greget, Fabien Pernot, Jean-Marie C. Bouteiller, Viviane Ghaderi, Sushmita Allam, Anne Florence Keller, Nicolas Ambert, Arnaud Legendre, Merdan Sarmis, Olivier Haeberle, Michel Faupel, Serge Bischoff, Theodore W. Berger, Michel Baudry

**Affiliations:** 1 Rhenovia Pharma, Mulhouse, France; 2 MIPS, Université de Haute Alsace, Mulhouse, France; 3 Neuroscience Program, University of Southern California, Los Angeles, California, United States of America; 4 Department of Biomedical Engineering, University of Southern California, Los Angeles, California, United States of America; Emory University, United States of America

## Abstract

Activation of several subtypes of glutamate receptors contributes to changes in postsynaptic calcium concentration at hippocampal synapses, resulting in various types of changes in synaptic strength. Thus, while activation of NMDA receptors has been shown to be critical for long-term potentiation (LTP) and long term depression (LTD) of synaptic transmission, activation of metabotropic glutamate receptors (mGluRs) has been linked to either LTP or LTD. While it is generally admitted that dynamic changes in postsynaptic calcium concentration represent the critical elements to determine the direction and amplitude of the changes in synaptic strength, it has been difficult to quantitatively estimate the relative contribution of the different types of glutamate receptors to these changes under different experimental conditions. Here we present a detailed model of a postsynaptic glutamatergic synapse that incorporates ionotropic and mGluR type I receptors, and we use this model to determine the role of the different receptors to the dynamics of postsynaptic calcium with different patterns of presynaptic activation. Our modeling framework includes glutamate vesicular release and diffusion in the cleft and a glutamate transporter that modulates extracellular glutamate concentration. Our results indicate that the contribution of mGluRs to changes in postsynaptic calcium concentration is minimal under basal stimulation conditions and becomes apparent only at high frequency of stimulation. Furthermore, the location of mGluRs in the postsynaptic membrane is also a critical factor, as activation of distant receptors contributes significantly less to calcium dynamics than more centrally located ones. These results confirm the important role of glutamate transporters and of the localization of mGluRs in postsynaptic sites in their signaling properties, and further strengthen the notion that mGluR activation significantly contributes to postsynaptic calcium dynamics only following high-frequency stimulation. They also provide a new tool to analyze the interactions between metabotropic and ionotropic glutamate receptors.

## Introduction

Glutamate is the main excitatory neurotransmitter in mammalian brain and mediates its effects through the activation of two major classes of receptors, the ionotropic (iGluRs) and metabotropic glutamate receptors (mGluRs). Ionotropic glutamate receptors consist of the α-amino-3-hydroxy-5-methyl-isoxazolepropionic acid receptors (AMPARs), N-methyl-D-aspartate receptors (NMDARs) and kainate receptors [1]. Glutamate metabotropic receptors belong to three subtypes of G-protein coupled receptors (GPCRs), mGluR type I (mGluRI), II and III, based on their sequence homology, pharmacology and signal transduction mechanisms. Activation of mGluRI receptors (mGluR_1_ and mGluR_5_) stimulates phospholipase C (PLC) to hydrolyze phosphatidylinositol 4,5-biphosphate (PIP2) in the cell plasma membrane leading to the formation of inositol 1,4,5-trisphosphate (IP3) and diacylglycerol (DAG) [2], [3]. While DAG stimulates protein kinase C (PKC), which is involved in various pathways, IP3 binds to a specific receptor on the endoplasmic reticulum (ER) membrane, resulting in calcium release and increase in cytosolic calcium concentration [4], [5], [6], [7], [8]. As activation of NMDA receptors (and possibly certain subtypes of AMPA receptors) also leads to calcium influx in postsynaptic structures, the precise contribution of mGluRI receptors to the dynamics of changes in spine calcium concentration is not well characterized and is difficult to assess at individual synapses. The calcium response is also dependent on the number and spatial distribution of the receptors, the synapse geometry and its location on the dendritic tree. In the CA1 region of hippocampus, mGluRI receptors are generally localized in the perisynaptic region of dendritic spines [9]. Because of this location, it has generally been assumed that glutamate released by a single presynaptic release event, which is subjected to diffusional dilution and reuptake, would not activate perisynaptic mGluRI. However, this receptor could become engaged at high frequency of synaptic activity [10], [11]. In addition, calcium dynamics in spines are regulated by complex interactions between calcium influx through a variety of calcium channels, calcium diffusion in various intracellular compartments, calcium pumps, various calcium binding proteins and intracellular stores. Considering the complexity of calcium homeostasis and the implication of mGluRI in various brain functions as well as disease states [12], [13], [14], there is a need to better understand the relative contribution of these mechanisms to calcium dynamics under different experimental conditions. Direct measurements of these parameters at a single synapse are extremely difficult, and experimental control over many synaptic mechanisms is still currently impossible, motivating the use of a biophysically realistic model of receptor activation at a single synapse. To better understand the role of mGluRI in calcium dynamics at the dendritic spine level, we developed and analyzed a detailed model of a glutamatergic synapse of the CA1 area of hippocampus incorporating the major elements involved in calcium regulation. In particular, the model includes AMPA and NMDA receptors and mGluRI receptors as well as a generic glutamate transporter to further assess the role of receptor location in calcium dynamics. Our results indicate that the contribution of mGluRI to calcium dynamics is dependent on both its synaptic localization and the stimulation frequency, and reveal interesting receptor interactions at the level of IP3 dynamics.

## Results

### Model of a CA1 glutamatergic synapse

**Figure 1:**
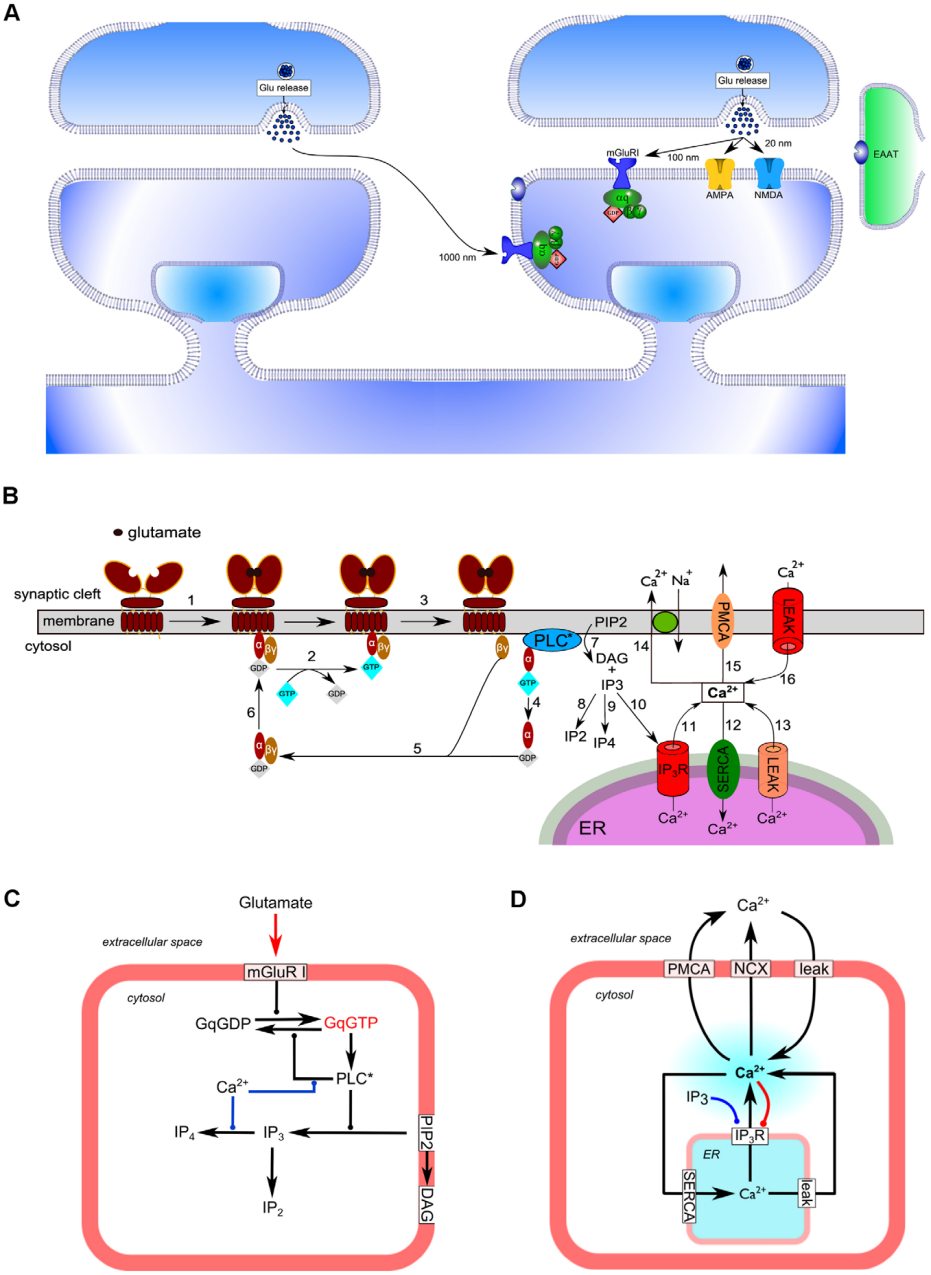
Schematic representation of the model of the glutamatergic synapse used in the study. (A) Overview of the global system incorporating glutamate release, glutamate diffusion, glutamate re-uptake by glutamate transporters (EAAT type, homogenously distributed starting at 200 nm from the release site), ionotropic glutamate receptors (AMPAR and NMDAR), and metabotropic glutamate receptors type I (mGluRI). Glutamate is released following presynaptic stimulation and diffuses in the synaptic cleft (Figure 4 and Methods). For simulation experiments, mGluRI was located at (i) 100 nm or alternatively at (ii) 1000 nm from glutamate release site (see Methods for further explanations). (B) Schematic of mGluRI model compartmentalization and reactions. (C) The mGluRI model (reactions 1–9 according to B): glutamate binds to mGluRI; activated mGluRI allows Gαq subunits liganded with GDP (Gq-GDP) to bind to an intracellular domain of the receptor, which facilitates GDP–GTP exchange. Gαq-GTP (GαGTP) binds and activates PLC (PLC*) in a Ca^2+^-dependent manner, which increases the GTPase activity of Gq-GTP. PLC* produces diacylglycerol (DAG) and inositol-3-phosphate (IP3) from phosphatidylinositol 4,5-bisphosphate (PIP2) in the inner membrane. IP3 is degraded into IP4 and IP2 by IP3 3-kinase and IP3 5-phosphatase, respectively. (D) Calcium dynamics model (reactions 10–16 according to B). IP3 binds to IP3 receptors (IP3R) on the endoplasmic reticulum (ER) membrane producing the release of Ca^2+^ into the cytosol. Cytosolic Ca^2+^ is pumped out by Na^+^/Ca^2+^ exchanger (NCX) and Ca^2+^-ATPase (PMCA) in the plasma membrane and by the sarco/endoplasmic reticulum Ca^2+^-ATPase (SERCA) in the endoplasmic reticulum (ER) membrane. Two leak mechanisms were added at the plasma and ER membranes. The model also includes binding of free cytosolic Ca^2+^ to buffers (not represented, see Methods). Calcium-dependent biochemical processes are indicated in red color.

Our modeling framework consists of a CA1 dendritic spine divided into four distinct compartments, i.e., cleft, post-synaptic density (PSD), cytosol and endoplasmic reticulum (ER), with volumes of 0.0015, 0.002, 0.02 and 0.002 µm^3^, respectively in accordance with the three dimensional structure of CA3-CA1 pyramidal cell synapses [15], [16] (Figure 1). The model incorporates AMPA and NMDA receptors as well as metabotropic glutamate receptors type I (mGluRI). It also includes the intracellular cascade activated by mGluRI, and a number of elements involved in calcium signaling (channels, pumps, leak channels). Synaptic release sites for glutamate were concentrated at the center of the presynaptic component, and consist in point source release of about 3,000 molecules of glutamate in the synaptic cleft. Given the potential location of mGluRI at various sites on dendritic spines of the CA1 area, we included glutamate diffusion and uptake in order to determine glutamate concentration at various locations following release. The glutamate diffusion model was based on a geometrical model [17]. Glutamate uptake was mediated by generic transporters distributed homogenously with a concentration of 0.5 mM within a radial segment of spine starting at 200 nm from the release site.

### Calibration of the mGluRI model

**Figure 2:**
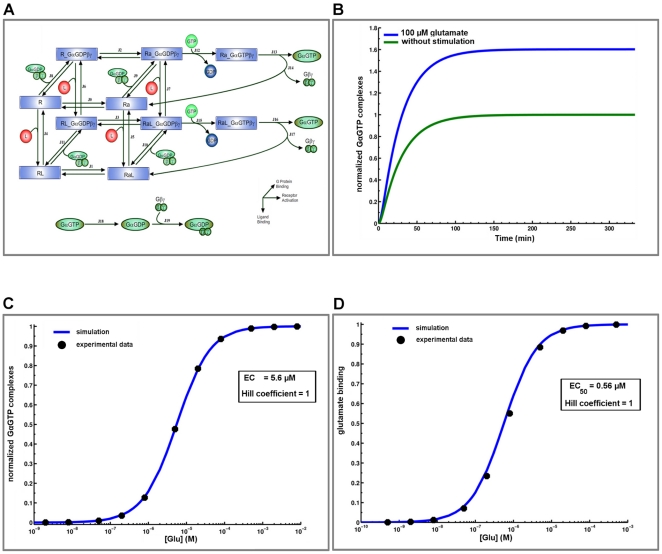
Calibration of the mGluRI model. (A) Kinetic scheme and rate equations describing interactions between glutamate, mGluRI and GαGTP formation were adapted from the ternary complex model described by Kinzer-Urserm [11]. Kinetic rates and equations are described in Table S1. The calculated activation time course of GαGTP formation (B) and concentration response curve (C) (blue line) were fitted to experimental [^35^S]-GTPγS binding data (black dots) [19]. With these kinetic parameters, the calculated concentration response curve of glutamate binding (D) provided an EC_50_ of 0.56 µM and a good fit with experimental data in [22].

The mGluRI model we used is a cubic ternary complex model derived from [18], and integrates a feedback loop for G-protein activation and recycling with dynamic receptor-G protein interactions (Figure 2A, Table S1). We used results from Akam et al. [19], who measured the accumulation of [^35^S]-GTPγS as a function of time and glutamate concentration, to calibrate the amplitude and dynamics of [Gα-GTP]. The kinetic rate constants of the model we derived from these data allowed to accurately reproduce the experimental results, including the amplitude and time course for [Gα-GTP] in the absence or presence (100 µM during 350 min) of glutamate (basal and active state, Figure 2B). Similarly, a long stimulation (140 min, Figure 2C) with increasing glutamate concentration generated simulated results that matched experimental data with an EC_50_ of 5.6 µM [19]. With this set of kinetic parameters, simulated glutamate binding concentration response matched various experimental data with an EC_50_ of 0.56 µM (Figure 2D) following long square applications of increasing glutamate concentration (10 s) [20], [21], [22].

### Calibration of IP3 receptor dynamics

The kinetic model we used for IP3 receptor dynamics (see Methods and Figure S1) was derived from previously validated computational models [23], [24]. As expected, the model accurately reproduced IP3 and calcium dependencies of the open probability of the receptor channel with a typical bell-shape curve (Figure S1).

### Calibration of mGluRI receptor-induced calcium dynamics in spine

**Figure 3:**
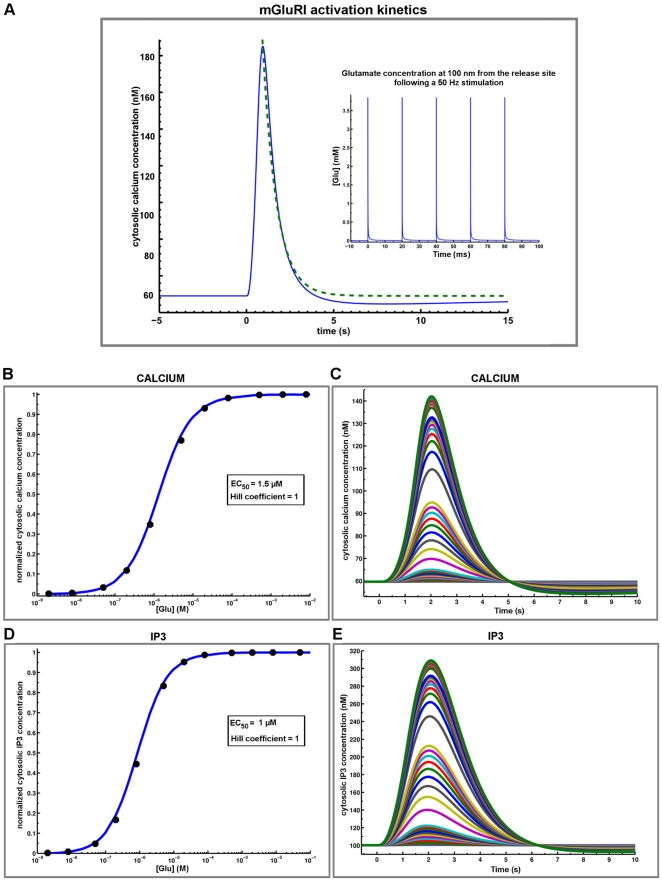
Calibration of mGluRI-mediated calcium dynamics in spine. (A) The calculated time course for cytosolic calcium concentration response curve (blue line) was fitted to experimental data (dashed green line) [18]. Using identical stimulation (inset, five applications of glutamate at 50 Hz), cytosolic calcium concentration was fitted with a decay time course of 800 ms. Ca^2+^ transient reached a peak amplitude of approximately 200 nM and lasted about 4 s. (B) With these kinetic parameters, the EC_50_ for mGluRI-mediated changes in calcium signal in cytosol (square pulse, 1 s) was 1.5 µM. Corresponding calcium dynamics following increasing glutamate concentrations are shown in C. (D) The EC_50_ for mGluRI-mediated changes in IP3 signal in cytosol (square pulse, 1 s) was 1.0 µM. Corresponding IP3 dynamics following increasing glutamate concentrations are shown in E.

The mGluRI signaling pathway was incorporated into the model of a CA1 dendritic spine, as described in Figure 1. To calibrate calcium transients induced by synaptic activation of mGluRI, we first performed a set of simulations under steady-state (equilibrium) condition (without any stimulus) in order to obtain the appropriate parameter values to generate resting calcium concentrations in cytosol, ER and extracellular compartments (see Methods). We then tested the effects of activation of mGluRI located at 100 nm opposite from the release site and fitted the parameters to reproduce the results from Schmidt and Eilers [25], who measured the amplitude and kinetics of calcium in a Purkinje cell dendritic spine, as the kinetics of spine calcium signals are similar in Purkinje and CA1 neurons [26]. Using identical stimulation parameters (five release events at 50 Hz, with mGluRI located at 100 nm from the release site), changes in cytosolic calcium concentration as a function of time were fitted with a mono-exponential decay curve with a τ value of 800 ms (Figure 3A). Calcium transients reached peak amplitude of approximately 200 nM and lasted about 4 s. The dynamics and amplitude of cytosolic calcium and IP3 signals following application of increasing glutamate concentration provided EC_50_ values of 1.5 µM and 1.0 µM, respectively (Figure 3B–3E), well within the range of experimental values (1–11 µM) [27].

### Incorporation of glutamate transporters in the spine environment

**Figure 4:**
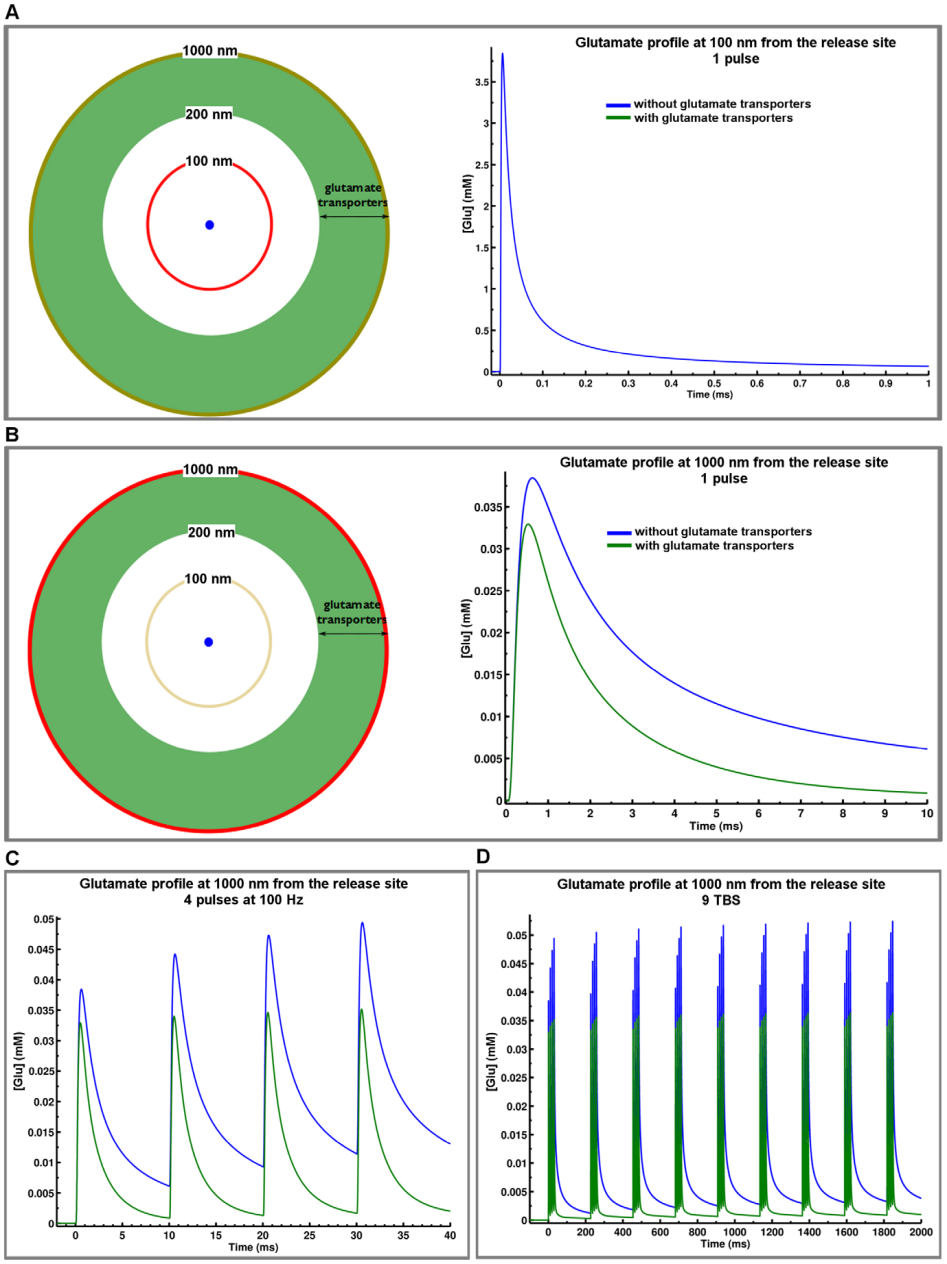
Influence of glutamate transporters on glutamate diffusion profile in the synaptic cleft. (A, B) Locations of simulated glutamate diffusion profiles are shown in projection (left side, red circle) relative to glutamate release (100 nm, A or 1000 nm, B). Time-course of glutamate concentration (right side) with (blue line) or without (green line) glutamate transporters in the model. Note the different scales for glutamate concentration at the two sites of analysis. (C, D) Glutamate transporters limit the summation of glutamate concentration at 1000 nm from the release site during one theta burst stimulation release protocol (TBS, four releases at 100 Hz, C) or 9 TBS (repetitive TBS with 200 ms delay between TBS, D).

A number of reports indicates that mGluRI are located mostly perisynaptically or even extrasynaptically. Considering the relatively low affinity of the receptors for glutamate, this localization suggests that glutamate uptake mechanisms could regulate their activation by controlling glutamate concentration available to “peri- and extrasynaptic” receptors. We tested this idea by incorporating a model of a generic glutamate transporter in the CA1 spine model with spatial characteristics discussed in Methods and in Figure 1 and Figure 4. We then examined the characteristics of extracellular glutamate concentration at different locations and following release evoked by various patterns of stimulation. We assumed that there were no transporters up to 200 nm away from the center of the postsynaptic density, and that transporters were uniformly distributed at distances >200 nm away from this center (Figure 4A, 4B and S2). Under these conditions, a single release of glutamate produced an increase in the local glutamate concentration reaching a maximum of 40 µM at 1000 nm from the release site as compared to 32 µM in the presence of glutamate transporters (Figure 4B). The presence of transporters also modified the decay time and amplitude of glutamate transients in the synaptic cleft following one single release (Figure 4B). The effects of glutamate transporters on glutamate concentration were amplified with repeated stimulation, as observed in response to a train of 4 pulses delivered at 100 Hz (Figure 4C), and even more so with a theta burst stimulation (TBS, 9 trains of 4 pulses at 100 Hz delivered at 5 Hz; Figure 4D). In the same manner, the presence of transporters limited the summation of glutamate concentration following an increase in stimulation frequency from 2 to 100 Hz (Figure S2). The limited summation of glutamate in the synaptic cleft during high frequency stimulation in the presence of glutamate transporters is in good agreement with their primary effect on decay time of glutamate diffusion rather than on peak amplitude (Figure 4).

At a distance of 1000 nm from the release site, the peak of glutamate concentration was about 30 µM, a value consistent with experimental results for extracellular glutamate in area CA1 in slices [28] (assuming that basal release in slices is generated by low spontaneous activity). Thus, the results indicate that the glutamate concentration profile at peri- and extrasynaptic sites is regulated by the presence of glutamate transporters. On the other hand, and consistent with experimental data [29], [30], [31] or those obtained with a different model [32], our simulations predicted no effect of transporters on the time course of glutamate within the synaptic cleft, which mainly reflects the rate of glutamate diffusion near the release site in our model.

### Impact of mGluRI location on calcium signals elicited by different stimulation protocols

**Figure 5:**
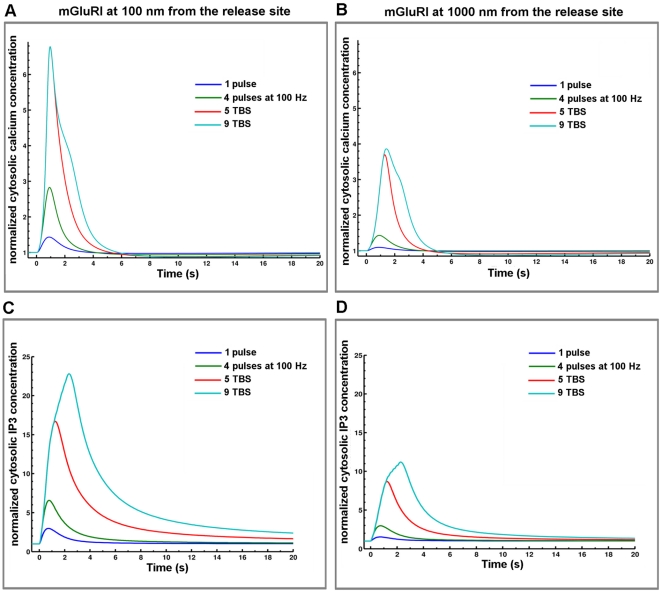
mGluRI-mediated changes in cytosolic calcium and IP3 concentrations for various stimulation patterns and different locations. (A, B) Temporal evolution of cytosolic calcium concentration generated by mGluRI activation in response to one release event, one burst (four releases at 100 Hz), 5 and 9 TBS (5 or 9 bursts with 200 ms delay between each) with mGluRI at 100 nm (A) or 1000 nm (B) from the release site. Peak response was reached with 5 TBS and increasing the number of TBS only modified decay kinetics. Calcium responses were normalized to the value of basal cytosolic calcium concentration (60 nM). (C, D) Temporal evolution of cytosolic IP3 concentration generated by mGluRI activation in response to the same patterns of stimulation as in A and B, with mGluRI at 100 nm (C) or 1000 nm (D) from the release site. IP3 responses were normalized to the value of basal cytosolic IP3 concentration (100 nM).

We next analyzed the impact of mGluRI location on IP3 and calcium dynamics in dendritic spines. In this case, we compared the patterns of responses elicited by activation of mGluRI located close to the center of the PSD (100 nm) or relatively far away (1000 nm), in order to also assess the potential effects of glutamate spillover resulting from neighboring synapse activation. Simulation results clearly indicated that the localization of the receptors had a profound impact on the magnitude and time-course of IP3 and calcium concentrations under various stimulation protocols (Figure 5). In particular, the results clearly indicated that the changes in IP3 and calcium concentrations elicited by a single stimulation were barely noticeable when the receptor was located at 1000 nm from the center of the PSD. Changes in these two parameters were greatly amplified with repeated stimulations, due to glutamate summation in the synaptic cleft, reaching a maximum for calcium with 5 bursts of stimulation delivered at 5 Hz (theta burst stimulation, TBS; one burst corresponding to four pulses at 100 Hz). Increasing the number of stimulation bursts to 9 modified the decay kinetics of cytosolic calcium concentration but not its amplitude (9 TBS, Figure 5A, 5B). On the other hand, IP3 formation did not reach its maximum with 5 TBS (Figure 5C, 5D). We also used tetanic stimulation instead of TBS, and in this case, there was no saturation of the calcium concentration after 50 pulses, and 1 sec stimulation at 100 Hz still increased peak and decay time of calcium concentration (Figure S3).

**Figure 6:**
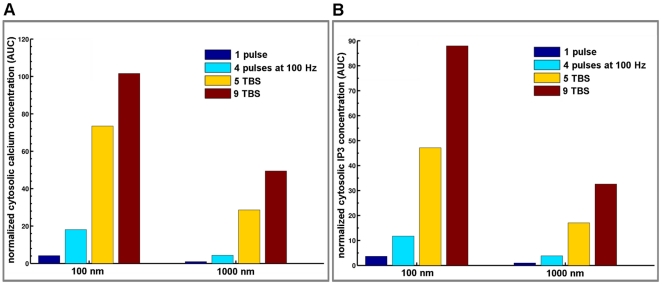
Effects of stimulation patterns and receptor location on integrated calcium and IP3 dynamics. (A) Histograms represent quantification of the area under the curve (AUC) for mGluRI-mediated calcium dynamics with various stimulation patterns and receptors located at 100 nm and 1000 nm from the release site. (B) Corresponding histograms of the AUC for mGluRI-mediated IP3 dynamics with various stimulation patterns and receptors located at 100 nm and 1000 nm from the release site. AUC values were normalized to the AUC value calculated for one release event with mGluRI located at 1000 nm from the release site.

To better illustrate the effects of glutamate release frequency and mGluRI location on calcium and IP3 kinetics, we calculated the areas under the curves (AUC) for cytosolic calcium or IP3 concentrations, which represent the integration over time of these parameters (Figure 6); in addition, it is possible that the integrated values for these two parameters are important for triggering various downstream signaling cascades and synaptic plasticity. For both calcium and IP3 concentrations, the pattern of changes in the integrated values was quite similar and underscored the large impact of the frequency of stimulation, as the normalized values of the AUCs increased by a factor of 25 with mGluRI located at 100 nm from the release site (9 TBS compared to one pulse). Qualitatively similar changes were observed whether mGluRI were localized at 100 or 1000 nm from the release site. The maximum values of the AUCs were still significantly lower with the receptors located far away from the release site, but revealed a greater impact of stimulation frequency, as the values for calcium and IP3 increased by factors of 50 and 32, respectively (9 TBS compared to one pulse). Similar effects were observed with tetanic stimulation at various frequencies, and the only differences were the higher values reached for AUCs at 100 Hz, which increased by factors of 33 and 41 for calcium and IP3, respectively with mGluRI located at 100 nm from the release site (Figure S3).

To further evaluate the role of glutamate transporters on the effects of the location of mGluRI on intracellular signaling, we analyzed changes in IP3 and calcium concentrations elicited by various stimulation frequencies in the absence or presence of transporters. Results of the simulations indicated that, independently of the frequency of stimulation, the presence of transporters entirely accounted for the differences observed at the two different locations (Figure S4).

### Incorporation of AMPA and NMDA receptors in the spine environment

**Figure 7:**
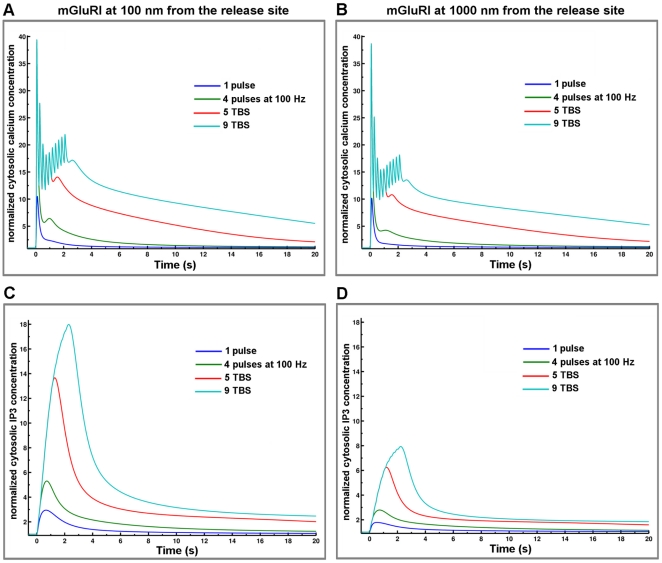
Effects of stimulation patterns and mGluRI location on glutamate-mediated changes in calcium and IP3 cytosolic concentrations. (A,B) Temporal evolution of cytosolic calcium concentration generated by glutamate receptor (AMPA, NMDA and mGluRI receptors) activation in response to one release event, one burst (four releases at 100 Hz), 5 and 9 TBS (repetitive bursts with 200 ms delay between each) with mGluRI located at 100 nm (A) or 1000 nm (B) from the release site. Note the summation effects and decay kinetics following 9 TBS. Calcium responses were normalized to the value of basal cytosolic calcium concentration (60 nM). (C, D) Temporal evolution of the cytosolic IP3 concentration generated by glutamate receptor (AMPA, NMDA and mGluRI receptors) activation in response to the same patterns of stimulation as in A and B with mGluRI located at 100 nm (A) or 1000 nm (B) from the release site. Note the summation effects and decay kinetics following 9 TBS. While mGluRI location has a very large impact on IP3 transients, it has only a weak impact on calcium dynamics. IP3 responses were normalized to the value of basal IP3 concentration (100 nM).

**Figure 8:**
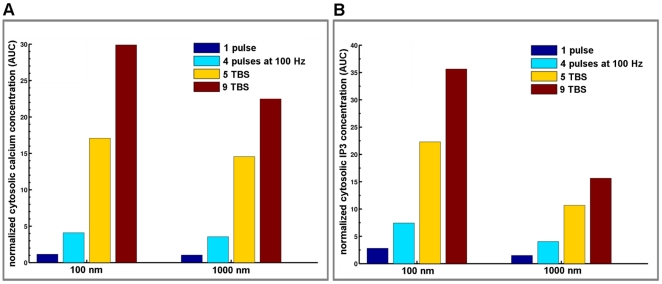
Effects of stimulation pattern and mGluRI location on integrated changes in calcium and IP3 concentration. (A) Histograms represent the quantification of the AUC for glutamate-mediated calcium dynamics with various stimulation patterns and mGluRI located at 100 nm and 100 nm from the release site. (B) Corresponding histograms of the AUC for glutamate-mediated IP3 dynamics with various stimulation patterns and mGluRI located at 100 nm and 1000 nm from the release site. AUC values were normalized to the AUC value calculated for one release event with mGluRI located at 1000 nm from the release site.

In addition to activating mGluRI, glutamate released from presynaptic terminals also activates ionotropic AMPA and NMDA glutamate receptors in dendritic spines. In order to study the contribution of mGluRI to calcium dynamics in spines following various patterns of synaptic activation, we incorporated kinetic models of AMPA and NMDA receptors in postsynaptic structures. The models of AMPA and NMDA receptors we used are described in the Methods Section and have been slightly modified [33] from those published in [34] and [35]. We then analyzed changes in IP3 and calcium concentrations elicited by various patterns of stimulation for 1 sec (Figure 7). Preliminary simulations indicated that AMPA and NMDA receptor-mediated responses were not modified by the presence of glutamate transporters (data not shown). To analyze the effect of stimulation frequency on calcium and IP3 signaling in dendritic spines, we applied bursts of glutamate release (1 pulse, 1 burst of 4 pulses at 100 Hz (4P), 5 bursts of 4 pulses at 100 Hz delivered at theta frequency (5 TBS) or 9 bursts at theta frequency (9 TBS) with mGluRI located at 100 nm or 1000 nm from the release site (Figure 7A–7D). While the location of mGluRI had a large effect on changes in IP3 formation (Figure 7C, 7D), it only had a minor effect on changes in calcium concentration in dendritic spines (Figure 7A, 7B). This was also evident when measuring AUC for both IP3 and calcium concentrations (Figure 8). As expected, increasing stimulation frequency resulted in very large increases in AUCs for both IP3 and calcium concentrations. However, the influence of the localization of mGluRI was relatively small and increased as a function of stimulation frequency (ratios at the values at 100 nm *vs.* 1000 nm were 1.10, 1.14, 1.17 and 1.18 following one release event, 4P, 5 TBS and 9 TBS respectively; Figure 8A). Thus, the addition of ionotropic receptors to the synapse environment decreased the impact of mGluRI location on postsynaptic calcium dynamics (100 nm or 1000 nm; compare Figures 6 and 8), although this effect is dependent on the stimulation frequency; at high frequency of stimulation, the effect of mGluRI location becomes more obvious. This result could be explained by (i) the increasing contribution of mGluRI to calcium signaling with stimulation frequency; (ii) an indirect effect through the downstream cascade of mGluRI as some elements of IP3 signaling are regulated by spine calcium concentration (see Methods and Figure 1), or (iii) a combination of these effects.

### Relative contribution of mGluRI to calcium dynamics in dendritic spines

**Figure 9:**
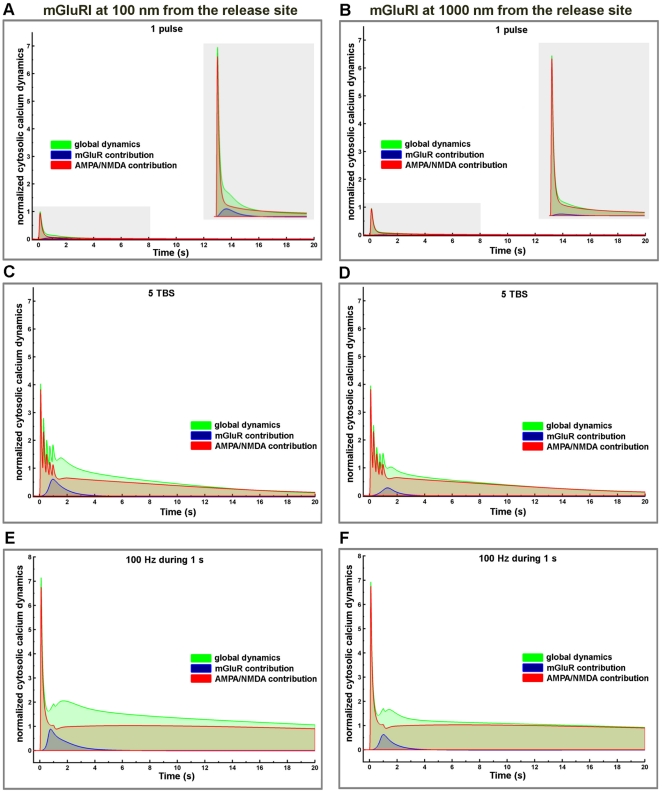
Respective contribution of ionotropic and metabotropic glutamate receptors on calcium cytosolic dynamics for different stimulation patterns and mGluRI locations. Temporal evolution of cytosolic calcium concentration dynamics (total response, green lines) generated by activation of ionotropic glutamate receptors (AMPA/NMDA, red lines) and mGluRI (blue lines) in response to one release event (A, B), 5 TBS (C,D) or 100 Hz during 1 s (E, F) with mGluRI located at 100 nm (A, C, E) or 1000 nm (B, D, F) from the release site. Values were normalized to the peak amplitude of the calcium response produced by AMPA, NMDA and mGluRI receptors combined, following one release event with mGluRI located at 100 nm from the release site (516 nM).

**Figure 10:**
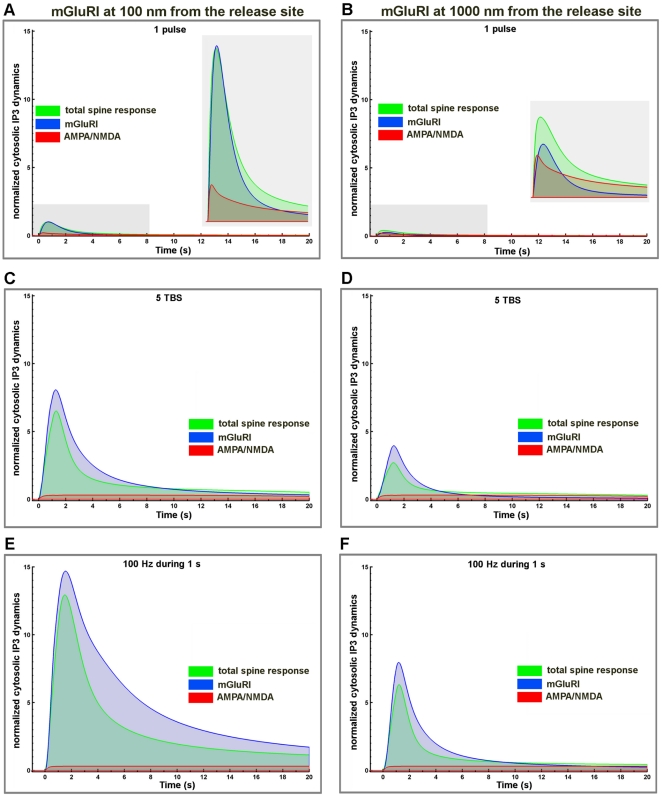
Respective contribution of ionotropic and metabotropic glutamate receptors on IP3 cytosolic dynamics for different stimulation patterns and mGluRI locations. Temporal evolution of cytosolic IP3 concentration (total response, green lines) generated by activation of ionotropic glutamate receptors (AMPA/NMDA, red lines) and mGluRI (blue lines) in response to one release event (A, B), 5 TBS (C,D) or 100 Hz during 1 s (E, F) with mGluRI located at 100 nm (A, C, E) or 1000 nm (B, D, F) from the release site. Values were normalized to the peak amplitude of the IP3 response produced by AMPA, NMDA and mGluRI receptors combined, following one release event with mGluRI located at 100 nm from the release site (315 nM).

**Figure 11:**
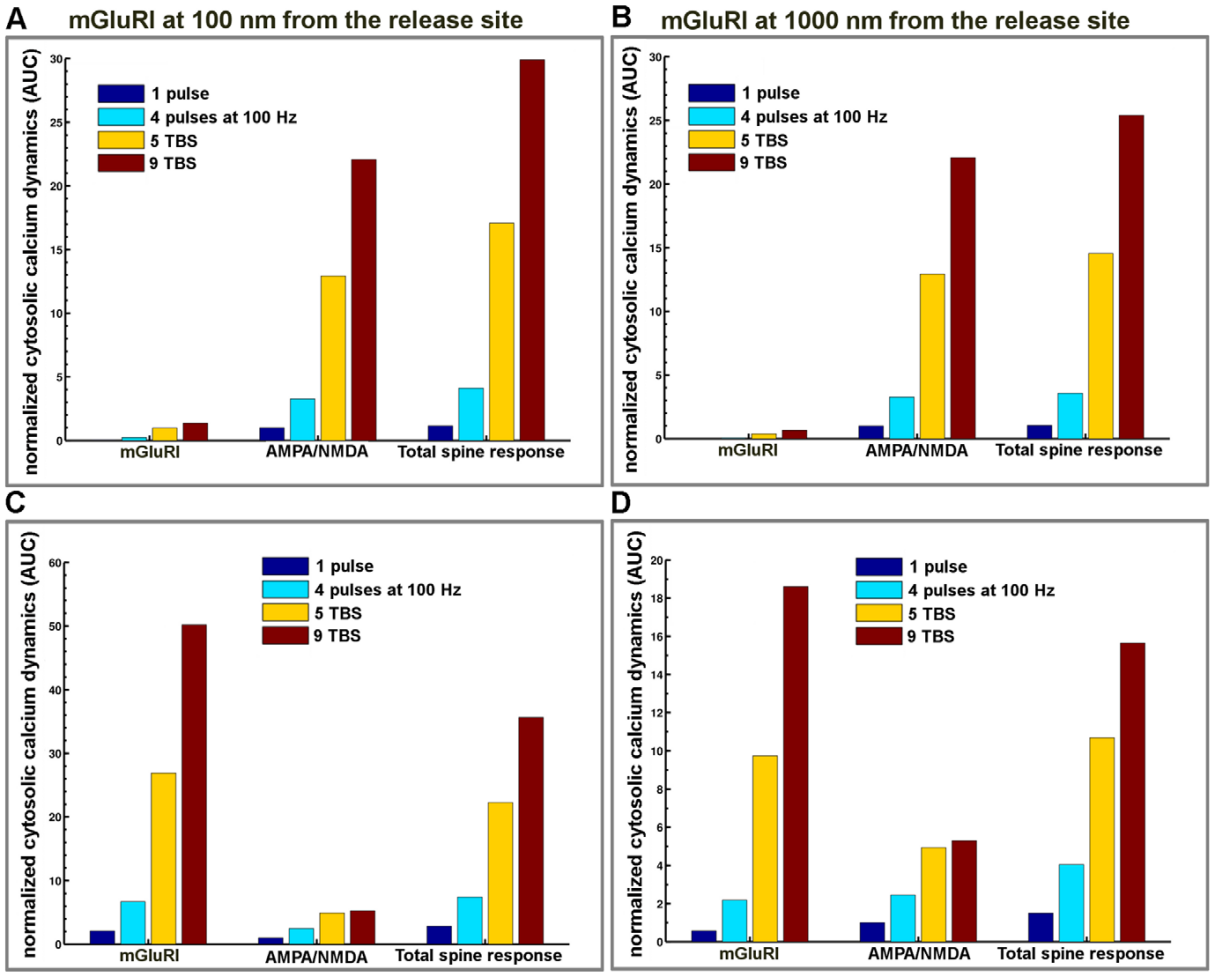
Interactions between ionotropic and metabotropic glutamate receptors in glutamate-mediated calcium and IP3 transients in dendritic spines. (A, B) Histograms represent the AUC for calcium dynamics generated by activation of mGluRI, ionotropic glutamate receptors (AMPA/NMDA), and all receptors combined (total spine response) with mGluRI located at 100 nm (A) or 1000 nm (B) from the release site following various stimulation patterns. Note the small, but significant supra-additive effect of the combined activation of all the receptors on calcium dynamics. (C, D) Corresponding histograms of the quantification of the AUC for IP3 dynamics generated by activation of mGluRI, ionotropic glutamate receptors (AMPA/NMDA), and all receptors combined (total spine response). Note the inhibitory effect of the activation of AMPA/NMDA receptors on the total response to glutamate stimulation, especially at high stimulation frequency. In all cases, AUC values were normalized to the AUC value calculated for one release event.

The respective contributions of mGluRI, AMPA and NMDA receptors to calcium dynamics were evaluated by superposing changes in calcium concentration produced by various patterns of stimulation (one release event, 5 TBS and 100 Hz during 1 s) with two different locations of mGluRI (100 nm and 1000 nm from the release site); in each case, the graphs include the contributions of mGluRI only, of AMPA/NMDA receptors, and the global dynamics (Figure 9). Overall, mGluRI contribution increased with stimulation frequency and produced a small modification of the decay rates. Similar results were obtained following tetanic stimulation at frequencies ranging from 1 Hz to 100 Hz (Figure S5). We also analyzed the relative contributions of mGluRI and AMPA/NMDA receptors on IP3 dynamics in dendritic spines (Figure 10), as both PLC and IP3 3-kinase activities are calcium-dependent [36], [37], [38]. The results clearly indicated that mGluRI activation contributed the large majority of IP3 dynamics, and that the relative contribution of AMPA/NMDA receptors was slightly larger when mGluRI localization was far from the release site (1000 nm), and the stimulation frequency low (Figure 10). Interestingly, AUC analysis of the respective contributions of metabotropic and ionotropic glutamate receptors to calcium and IP3 changes revealed significant non-linear interactions between ionotropic and metabotropic receptors, irrespective of mGluRI location (Figure 11A–11D). In particular, whereas a supra-additive effect on calcium dynamics was produced by activation of all glutamate receptors, activation of ionotropic receptors reduced the effects of mGluRI activation of IP3 dynamics, which was more pronounced at high frequency of stimulation. Similar effects were observed with tetanic stimulation at various frequencies (Figure S6). Clearly, these results demonstrated that mGluRI contribution to calcium signaling is dependent of the complex combination of calcium influx induced by ionotropic glutamate receptors, calcium-dependent IP3 mobilization and metabolism, and mGluRI location.

**Figure 12:**
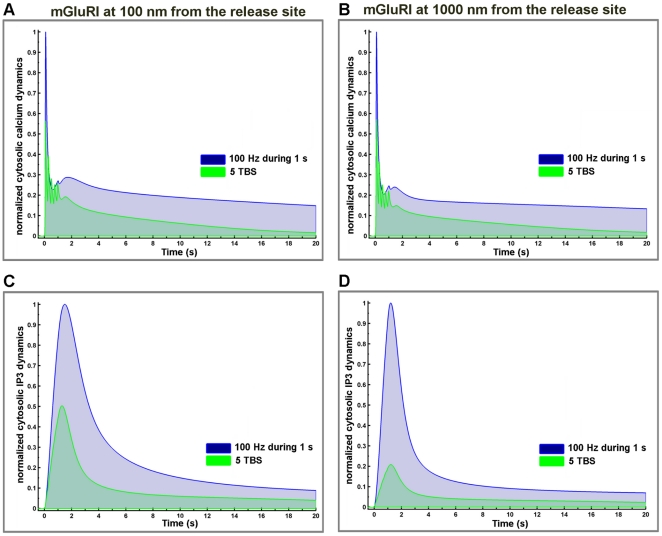
Comparison of the effects of tetanic stimulation *vs.* TBS on changes in calcium and IP3 concentration. (A, B) Temporal evolution of cytosolic calcium dynamics generated by a 1-s stimulation with 5 TBS (green line) or 100 Hz frequency (blue line) with mGluRI located at 100 nm (A) or 1000 nm (B). Values were normalized to the value observed with TBS. (C, D) Corresponding temporal evolution of cytosolic IP3 dynamics with mGluRI located at 100 nm (C) or 1000 nm (D). Values were normalized to the peak amplitude response produced by AMPA, NMDA and mGluRI receptors combined following the 100 Hz tetanic stimulation protocol for calcium (3.3 µM) and IP3 (1.4 µM).

Finally, as various laboratories use tetanic stimulation at 100 Hz for 1 sec or TBS to elicit changes in synaptic strength, we directly compared calcium and IP3 dynamics following 1 s of tetanic pulses at 100 Hz and 5 TBS (Figure 12). The results clearly indicated that the 100 Hz-stimulation resulted in much higher levels of intracellular IP3 and calcium concentrations in the postsynaptic spine than the 5 TBS protocol. More importantly, they also showed that the duration of calcium and IP3 signals were significantly prolonged with tetanic stimulation as compared to the TBS protocol.

## Discussion

The glutamatergic synapse model we have developed, incorporating both ionotropic and metabotropic receptors as well as glutamate transporters, is the first computational model to provide a qualitative and quantitative analysis of glutamatergic neurotransmission. This model permits the analysis of the respective contributions of glutamate receptors to changes in intracellular calcium and IP3 concentrations in dendritic spines as a function of time following various patterns of synaptic stimulation. This model also provided quantitative information regarding the effect of mGluRI localization and glutamate transporters on the postsynaptic dynamics of calcium and IP3 concentration profiles. In agreement with a previous report [39], our results clearly demonstrated that the location of mGluRI has a large impact on its activation, as the presence of glutamate transporters significantly reduces the availability of glutamate at sites distant from the release site. Our results also underscored the frequency dependency of mGluRI activation and clearly showed that mGluRI-mediated changes in IP3 and the resulting increase in intracellular calcium concentration are minimal at low frequency of synaptic activation and become significant only at high frequency of stimulation, and in particular with tetanic stimulation. Interestingly, both the role of the localization of mGluRI and its frequency dependency are accounted for by the presence of glutamate transporters that significantly reduce extracellular glutamate concentration at low frequency of presynaptic release, and more so at high frequency of release [40], [41]. These results are also consistent with experimental evidence indicating that pharmacological inhibition of glutamate transporters greatly enhanced mGluRI-mediated responses [42]. Kinetic properties of mGluRI could also account for the stimulus frequency-dependency of mGluRI-mediated responses, in agreement with recent experimental data [43].

Interestingly, our results indicated the existence of non-linear interactions between ionotropic and mGluRI receptors on the dynamics of cytosolic calcium and IP3 concentration, irrespective of the localization of mGluRI. Thus, while the combined activation of the receptors produced a synergistic effect on calcium dynamics, it resulted in a decreased production of IP3, as compared to activation of mGluRI alone, especially with high frequency of stimulation, due to calcium-dependent mechanisms of IP3 metabolism. These non-obvious results are critically due to the calcium dependency of various enzymatic activities, in particular IP3-3 kinase activity. Notably, the strong impact of NMDA receptor activation on spine calcium concentration stimulates the degradation of mGluRI-induced IP3 formation. The functional significance of this phenomenon is not clear at the moment, but it could represent a feed-back mechanism to limit the degradation of membrane phospholipids at high frequency of stimulation, which could be detrimental for membrane functions. Finally, our results also indicate that tetanic stimulation produces much larger and longer changes in calcium concentration than TBS protocols. Such a difference could account for the different types of synaptic plasticity triggered by these two stimulation protocols [44].

The potential contribution of mGluRI in LTP induction in various brain regions has been the subject of numerous reports, although the exact role of these receptors in various forms of LTP remains unclear [45]. A number of studies suggest that mGluRI activation facilitates the induction of NMDA receptor-dependent LTP. According to our results, the contribution of mGluRI to postsynaptic calcium dynamics remains negligible even at high frequency of stimulation and with receptors present close to the center of the postsynaptic density, as compared to that of the ionotropic receptors. However, it is possible that activation of mGluRI by prolonged exogenous application of agonists results in significant changes in postsynaptic calcium concentration, which could lead to changes in synaptic efficacy, as has been repeatedly reported [46], [47]. In addition, it is important to stress that a recent report indicates that, in hippocampus, endoplasmic reticulum is present mostly in large dendritic spines, suggesting that mGluRI activation is likely to modify synaptic function only at a subset of hippocampal synapses [48]. Finally, recent results implicate mGluRI in LTP in hippocampal interneurons [49], and it would be interesting to perform similar simulations to those we have done here at glutamatergic synapses on interneurons, which lack dendritic spines.

Similarly, the role of mGluRI activation in LTD induction, while extensively discussed, remains not completely elucidated. Several mechanisms have been proposed to account for mGluRI-dependent LTD. One such mechanism is clearly calcium-independent and appears to depend on interactions between the C-terminal domain of mGluRI and intracellular proteins such as Homer and downstream cascades [50]. However, in cerebellum, it appears that mGluRI-dependent LTD requires calcium release from intracellular stores, and results in internalization of AMPA receptors [51]. While we did not attempt to model a glutamatergic synapse in cerebellum, our model could relatively easily be adapted to this synapse and provide interesting information regarding the temporal profile of calcium concentration elicited by stimulation protocols leading to LTD at cerebellar synapses.

Ultimately, our model could be used to accurately describe how pharmacological or pathological conditions could modify glutamatergic transmission, and to identify potential molecular targets that could be experimentally evaluated. Because of the wide diversity, heterogeneous distribution, and diverse physiological roles of mGluR subtypes, there has been a large effort to identify pharmacological compounds capable of modulating selective subtypes of mGluRs in order to develop new therapeutic treatments for a number of CNS diseases. Such drugs could have a significant impact in the treatment of a variety of psychiatric and neurological disorders including depression, anxiety disorders, schizophrenia, chronic pain, epilepsy, Alzheimer's disease, and Parkinson's disease. In particular, mGluR5 receptors are closely associated with NMDA receptors and might play a significant role in setting the tone of NMDA receptor function in forebrain regions containing neuronal circuits important for cognitive behavior. A better understanding of the interactions between mGluR5 and NMDA receptors would clearly facilitate the development of better strategies to treat diseases associated with learning disorders. Our detailed computational model can be used to simulate these various pathological conditions and shed some light on the strategic impact and quantitative evaluation of targeting mGluRI in drug discovery process. This model is integrated in a more complete numerical glutamatergic synapse that allows access to a large number of read-outs difficult to analyze experimentally in a quantitative way, and potentially the discovery of synergistic actions of drug combinations.

## Methods

### The mGluRI activation model

The model is a cubic ternary complex activation model [18] that integrates a feedback loop of G protein activation and recycling with dynamic ligand-receptor-G protein interactions (Figure 2A, Table S1). The key output of the model, [G_α_GTP], was normalized to the dynamic value of [G_α_GTP] under basal conditions (without glutamate stimulation, Figure 2). Concentration-response curves were generated by calculating the maximum equilibrium values of [G_α_GTP] following glutamate stimulation at various concentrations. Calibration of the model was done with experiments measuring the accumulation of radiolabeled G_α_GTP (Figure 2B, 2C). G protein activation induced IP3 production via phospholipase C activation, and cytosolic IP3 was degraded by calcium-dependent IP3 3-kinase and IP3 5-phosphatase.

### The IP3R activation model

We incorporated kinetic properties of IP3R channels that release calcium from the ER. The IP3R model was derived from the original De Young-Keizer conceptualization [23] (Figure S1 and Table S1). The IP3R model reproduced the open probability of IP3R at equilibrium, and exhibited a bell-shaped Ca^2+^ dependency with a peak free cytosolic Ca^2+^ concentration of 0.2– 0.5 µM [52], [53].

### The calcium dynamics model

We constructed a diagram of calcium homeostasis (Figure 1C–D) based on a conceptual model [54] and adapted to a CA3-CA1 synapse [55]. Cytosolic calcium is pumped across the plasma membrane by Na^+^/Ca^2+^ exchanger (NCX), Ca^2+^-ATPase (PMCA), and a leak channel, and across the endoplasmic reticulum (ER) by sarcoplasmic/endoplasmic reticulum Ca^2+^-ATPase (SERCA) and a leak channel. Free calcium binds to calmodulin, calcineurin and protein kinase C (PKC) in cytosol and PSD. We also assumed that, because of the small volume of the spine, the molecules it contains are homogeneously distributed, and that their concentrations are uniform within each compartment. Spatial diffusion of molecules between compartments is mediated by channels and pumps, except for the diffusion of IP3 and Ca^2+^ between the cytosol and the PSD. Under baseline conditions, five mGluRI (equivalent to 8 µM, cf. [36]) were clustered within the perisynaptic zone at 100 nm from the release site. An alternative location of mGluRI was chosen at 1000 nm from the release site to evaluate the impact of mGluRI location on signaling. We modeled AMPA and NMDA receptors with detailed kinetics models based on a previous study of our group [33]. Under baseline conditions, 80 AMPARs and 20 NMDARs were clustered within the PSD at 20 nm from the release site and their kinetics were fitted with experimental data [56], [57] (note that we verified that the location of AMPA and NMDA receptors within the postsynaptic density did not modify the kinetics of calcium and IP3 concentrations (data not shown)). Calcium was assumed to diffuse from the PSD to the cytosol and to regulate some critical mechanisms: (i) PLC [58]; (ii) IP3-3 kinase [53] and (iii) IP3 receptor activities [24], [52]. Reaction equations, rate laws, and kinetic parameters of the global synapse model (mGluR activation, IP3 formation, IP3R activation and calcium dynamics) were added in Table S1.

### Simulations and analysis of the model

We modeled the dynamics of the system with ordinary differential equations for all variables according to the law of mass action, and the outputs of the system represent the solution of the system of differential equations. Model outputs were compared with existing experimental data, when available. The baseline was determined at equilibrium in the absence of glutamate release. All simulations were initiated from this baseline. Cytosolic calcium and IP3 at baseline converged to 60 and 100 nM, respectively. Calcium concentration in the synaptic cleft and ER converged to 2 and 0.5 mM, respectively. Temporal responses in cytosolic calcium and IP3 concentrations under various stimulation patterns and with different locations of mGluRI (Figure 5) and including AMPA and NMDA receptor dynamics (Figure 7) were normalized to the value of basal cytosolic calcium or IP3 concentration, respectively. To better illustrate the temporal integration of calcium and IP3 kinetics, we calculated the areas under the curves (AUC) using Riemann sum, a method for approximating the total area underneath a curve on a graph equivalent to an integral. The AUC results were normalized for mGluRI stimulation using AUC value obtained by mGluRI alone located at 1000 nm stimulated by one release event (Figure 6). When we added AMPA and NMDA receptor models, AUC values were normalized using AUC value obtained for AMPA and NMDA receptor responses following one release event (Figure 8). As AMPA and NMDA receptors were fixed at 20 nm from the release site, this normalization process allows a direct comparison with mGluRI location effects (Figure 11). Simulations were optimized and calibrated out using a personal computer and Matlab software (Mathworks Inc., Natick, MA, USA). This metamodel was implemented into a further detailed synapse platform, RHENOMS™ and simulated on a cluster nodes.

## Supporting Information

Figure S1 **Detailed IP3 receptor kinetic model and its validation.** (A) Kinetic model of the IP3 receptor (IP3R) incorporating calcium and IP3 binding and the open state (adapted from [23], [24], [55]). The model is composed of four independent identical subunits with an inhibitory Ca^2+^ binding site. Both IP3 and Ca^2+^ are required for receptor/channel opening. (B) Calculated open probability of IP3R as a function of cytosolic Ca^2+^ concentration (x-axis) and for a wide range of IP3 concentration (colored lines). (C) 3D representation of the open probability of IP3R (z-axis) as a function of Ca^2+^ and IP3 concentrations (x- and y-axis).
(TIFF)

Figure S2 **Influence of glutamate transporters on extrasynaptic glutamate concentration (1000 nm from the release site).** Changes in extrasynaptic glutamate concentration in the presence (green line) or absence (blue line) of glutamate transporters with presynaptic stimulation frequency of 2 Hz (A), 10 Hz (B), 50 Hz (C) and 100 Hz (D). Note that the presence of glutamate transporters significantly limits glutamate accumulation at 1000 nm from the release site, especially at high stimulation frequencies.
(TIFF)

Figure S3 **Effects of stimulation frequency and localization on mGluRI-mediated calcium and IP3 dynamics.** (A, B) Temporal evolution of cytosolic calcium concentration generated by mGluRI activation in response to increasing release frequency (color lines) with mGluRI at 100 nm (A) or 1000 nm (B) from the release site. Calcium responses were normalized to the value of basal cytosolic calcium concentration (60 nM). (C, D) Temporal evolution of cytosolic IP3 concentration generated by mGluRI activation in response to increasing release frequency with mGluRI at 100 nm (C) or 1000 nm (D) from the release site. IP3 responses were normalized to basal IP3 concentration (100 nM). (E, F) Histograms represent the area under the curve (AUC) of mGluRI-mediated calcium (E) or IP3 (F) dynamics in response to increasing release frequency with mGluRI located at 100 nm or 1000 nm from the release site. AUC values were normalized against AUC signal corresponding to response produced by AMPA and NMDA receptors following one release event for calcium and IP3 transients. Note that AUC of cytosolic calcium response produced by mGluRI following a 20 Hz stimulation protocol corresponds to the AUC of cytosolic calcium response produced by AMPA/NMDA receptors following one release event.
(TIFF)

Figure S4 **Effects of stimulation frequency, glutamate transporters, and mGluRI location on glutamate-mediated calcium and IP3 dynamics.** (A, B) Temporal evolution of cytosolic calcium (A) and IP3 (B) concentrations generated by glutamate receptor (AMPA, NMDA and mGluRI receptors) activation in response to various stimulation frequencies (color lines) with mGluRI receptors located at 100 nm from the release site. (C, D) Corresponding temporal evolution of cytosolic calcium (C) and IP3 (D) concentrations generated by glutamate receptor (AMPA, NMDA and mGluRI receptors) activation in response to various stimulation frequencies (color lines) with mGluRI receptors located at 1000 nm (B) from the release site in the presence of glutamate transporters. Note the small effect of the location of mGluRI on calcium dynamics as opposed to the large effect on IP3 dynamics. (E, F) Temporal evolution of cytosolic calcium (E) and IP3 (F) concentrations generated by glutamate receptor (AMPA, NMDA and mGluRI receptors) activation in response to various stimulation frequencies (color lines) with mGluRI receptors located at 1000 nm (B) from the release site in the absence of glutamate transporters. Note that absence of glutamate transporters reversed the effects of the localization of mGluRI away from the release site for both calcium and IP3 dynamics. Calcium and IP3 responses were normalized to respective cytosolic basal concentrations (60 and 100 nM).
(TIFF)

Figure S5 **Effects of stimulation frequency and mGluRI location on the respective contribution of ionotropic and metabotropic glutamate receptors on integrated calcium and IP3 dynamics.** (A, B) Histograms represent the AUC for calcium (A) and IP3 (B) dynamics generated by activation of mGluRI and ionotropic glutamate receptors (AMPA/NMDA) with mGluRI located at 100 nm or 1000 nm from the release site at various stimulation frequencies. AUC values were normalized to the AUC value calculated for AMPA/NMDA response following one release event.
(TIFF)

Figure S6 **Interactions between ionotropic and metabotropic receptors in glutamate-mediated calcium and IP3 transients in dendritic spines.** (A, B) Histograms represent the quantification of the AUC for calcium dynamics generated by activation of mGluRI, ionotropic glutamate receptors (AMPA/NMDA), and all receptors (total spine response) with mGluRI located at 100 nm (A) or 1000 nm (B) from the release site at various stimulation frequencies. Note the significant supra-additive effect of the combined activation of all the receptors on calcium dynamics. (C, D) Corresponding histograms of the quantification of AUC for IP3 dynamics generated by activation of mGluRI, ionotropic glutamate receptors (AMPA/NMDA), and all receptors (total spine response) with mGluRI located at 100 nm (C) or 1000 nm (D) from the release site at various stimulation frequencies. Note the inhibitory effect of the activation of AMPA/NMDA receptors on the total response to glutamate stimulation, especially at high stimulation frequency. In all cases, AUC values were normalized to the AUC value calculated for AMPA/NMDA response following one release event.
(TIFF)

Table S1 **Reaction equations, rate laws, and kinetic parameters of the mGluRI and calcium dynamics models.**
(DOC)
