# Supplementary figures and images for: Simulation of Postsynaptic Glutamate Receptors Reveals Critical Features of Glutamatergic Transmission
Source: PLoS One. 2011 Dec 15;6(12):e28380. doi: 10.1371/journal.pone.0028380 (PMC3240618; doi:10.1371/journal.pone.0028380)

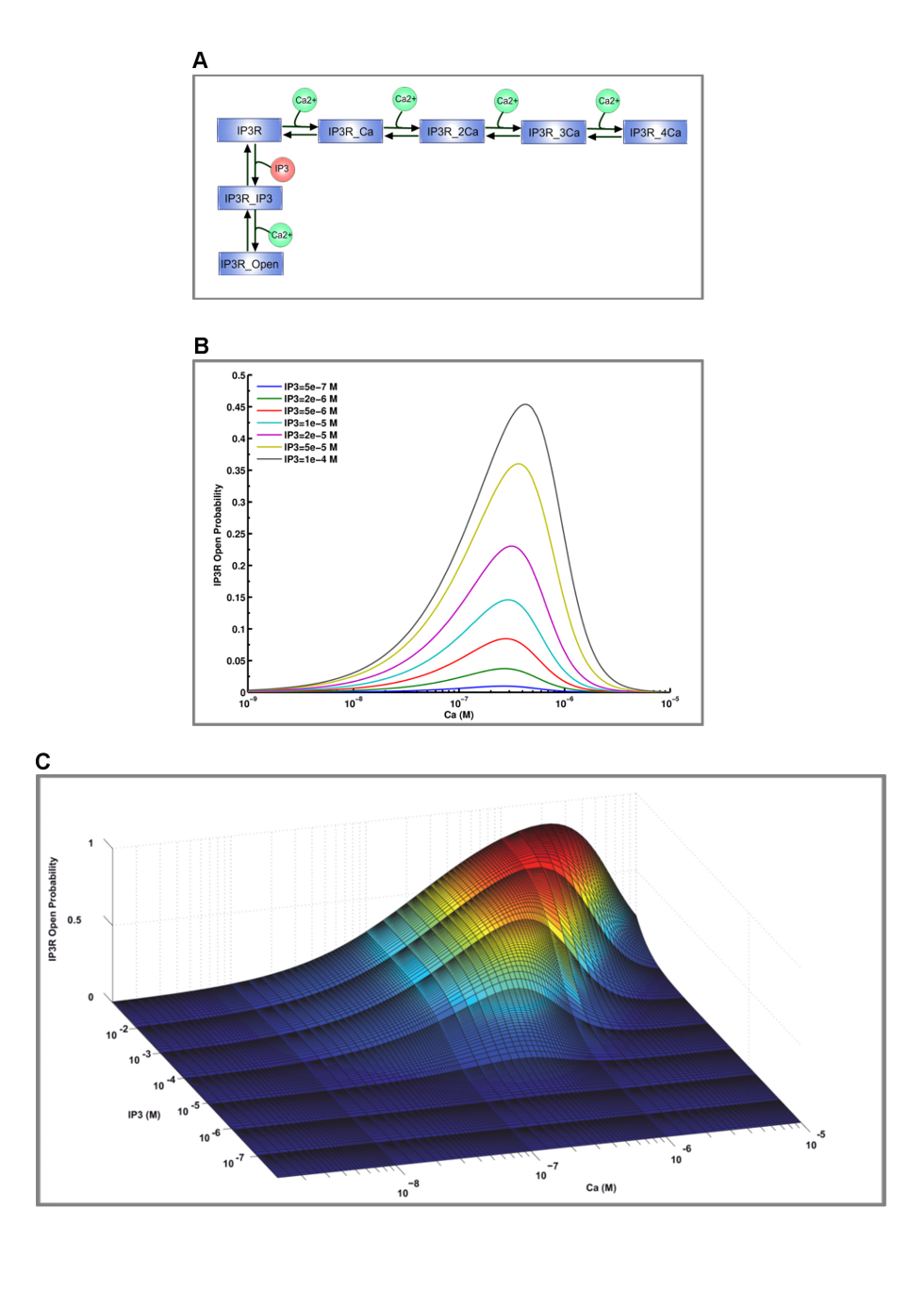

Supplement: Figure S1 — Detailed IP3 receptor kinetic model and its validation. (A) Kinetic model of the IP3 receptor (IP3R) incorporating calcium and IP3 binding and the open state (adapted from [23], [24], [55]). The model is composed of four independent identical subunits with an inhibitory Ca2+ binding site. Both IP3 and Ca2+ are required for receptor/channel opening. (B) Calculated open probability of IP3R as a function of cytosolic Ca2+ concentration (x-axis) and for a wide range of IP3 concentration (colored lines). (C) 3D representation of the open probability of IP3R (z-axis) as a function of Ca2+ and IP3 concentrations (x- and y-axis). (TIFF) [file pone.0028380.s001.tiff]

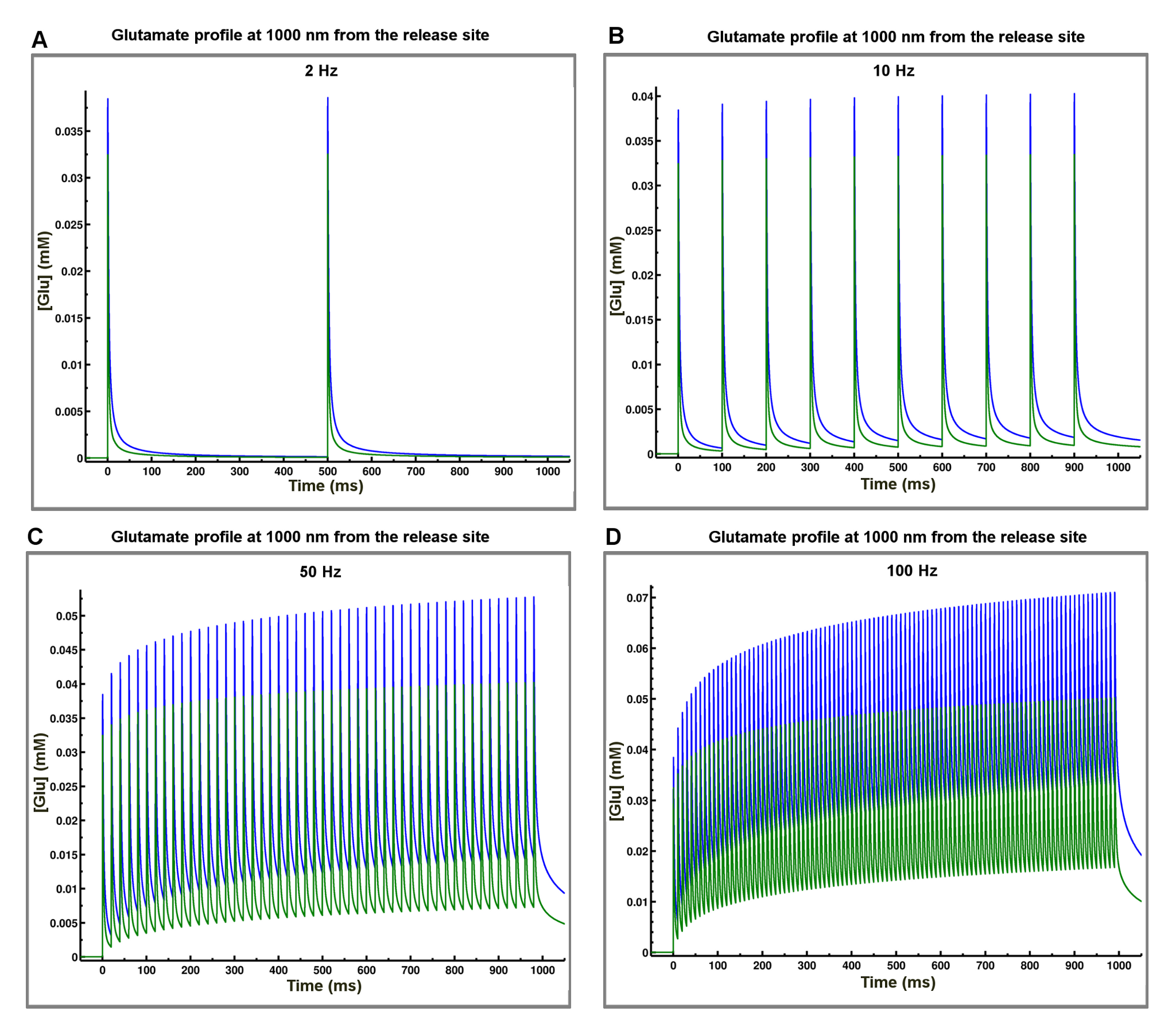

Supplement: Figure S2 — Influence of glutamate transporters on extrasynaptic glutamate concentration (1000 nm from the release site). Changes in extrasynaptic glutamate concentration in the presence (green line) or absence (blue line) of glutamate transporters with presynaptic stimulation frequency of 2 Hz (A), 10 Hz (B), 50 Hz (C) and 100 Hz (D). Note that the presence of glutamate transporters significantly limits glutamate accumulation at 1000 nm from the release site, especially at high stimulation frequencies. (TIFF) [file pone.0028380.s002.tiff]

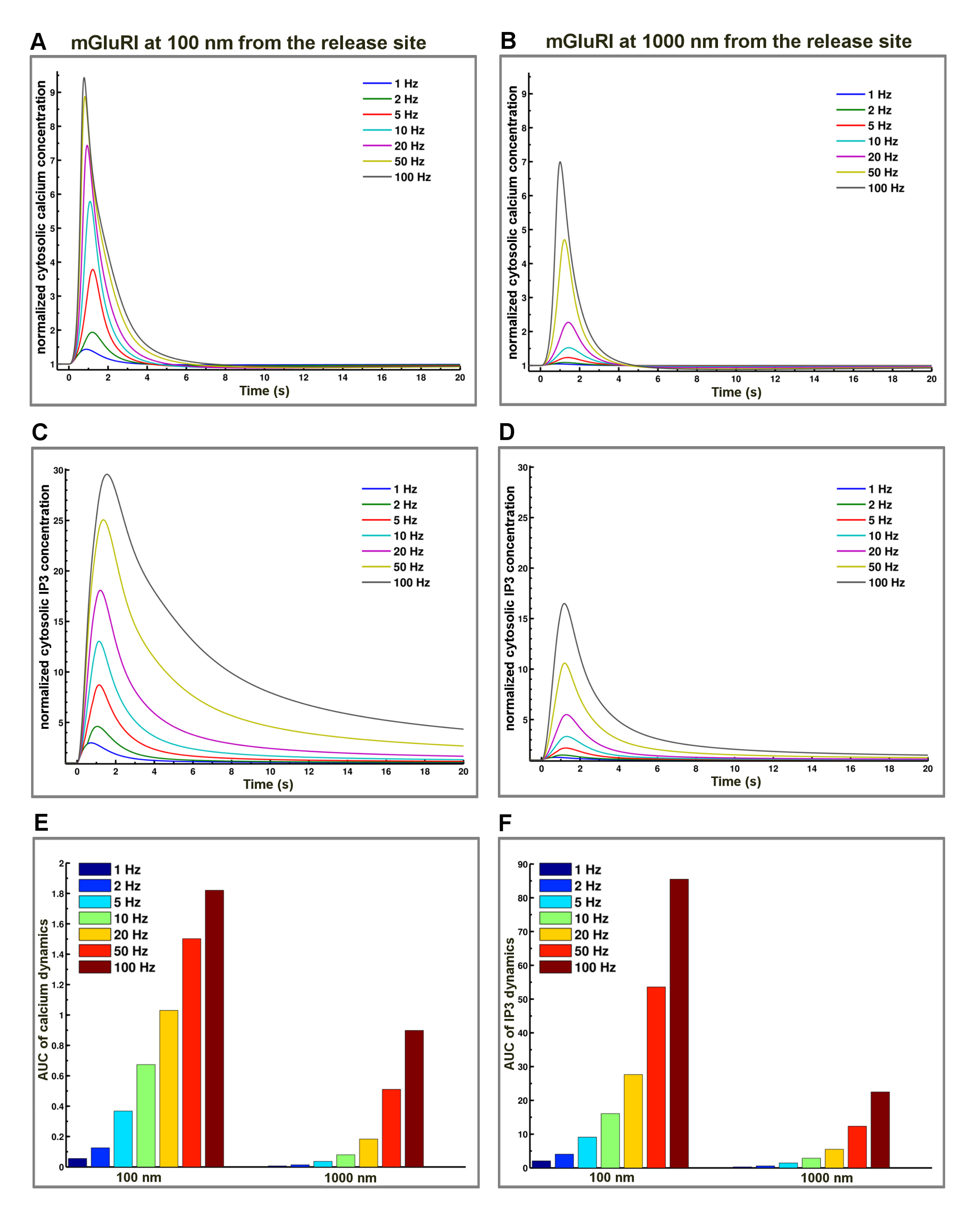

Supplement: Figure S3 — Effects of stimulation frequency and localization on mGluRI-mediated calcium and IP3 dynamics. (A, B) Temporal evolution of cytosolic calcium concentration generated by mGluRI activation in response to increasing release frequency (color lines) with mGluRI at 100 nm (A) or 1000 nm (B) from the release site. Calcium responses were normalized to the value of basal cytosolic calcium concentration (60 nM). (C, D) Temporal evolution of cytosolic IP3 concentration generated by mGluRI activation in response to increasing release frequency with mGluRI at 100 nm (C) or 1000 nm (D) from the release site. IP3 responses were normalized to basal IP3 concentration (100 nM). (E, F) Histograms represent the area under the curve (AUC) of mGluRI-mediated calcium (E) or IP3 (F) dynamics in response to increasing release frequency with mGluRI located at 100 nm or 1000 nm from the release site. AUC values were normalized against AUC signal corresponding to response produced by AMPA and NMDA receptors following one release event for calcium and IP3 transients. Note that AUC of cytosolic calcium response produced by mGluRI following a 20 Hz stimulation protocol corresponds to the AUC of cytosolic calcium response produced by AMPA/NMDA receptors following one release event. (TIFF) [file pone.0028380.s003.tiff]

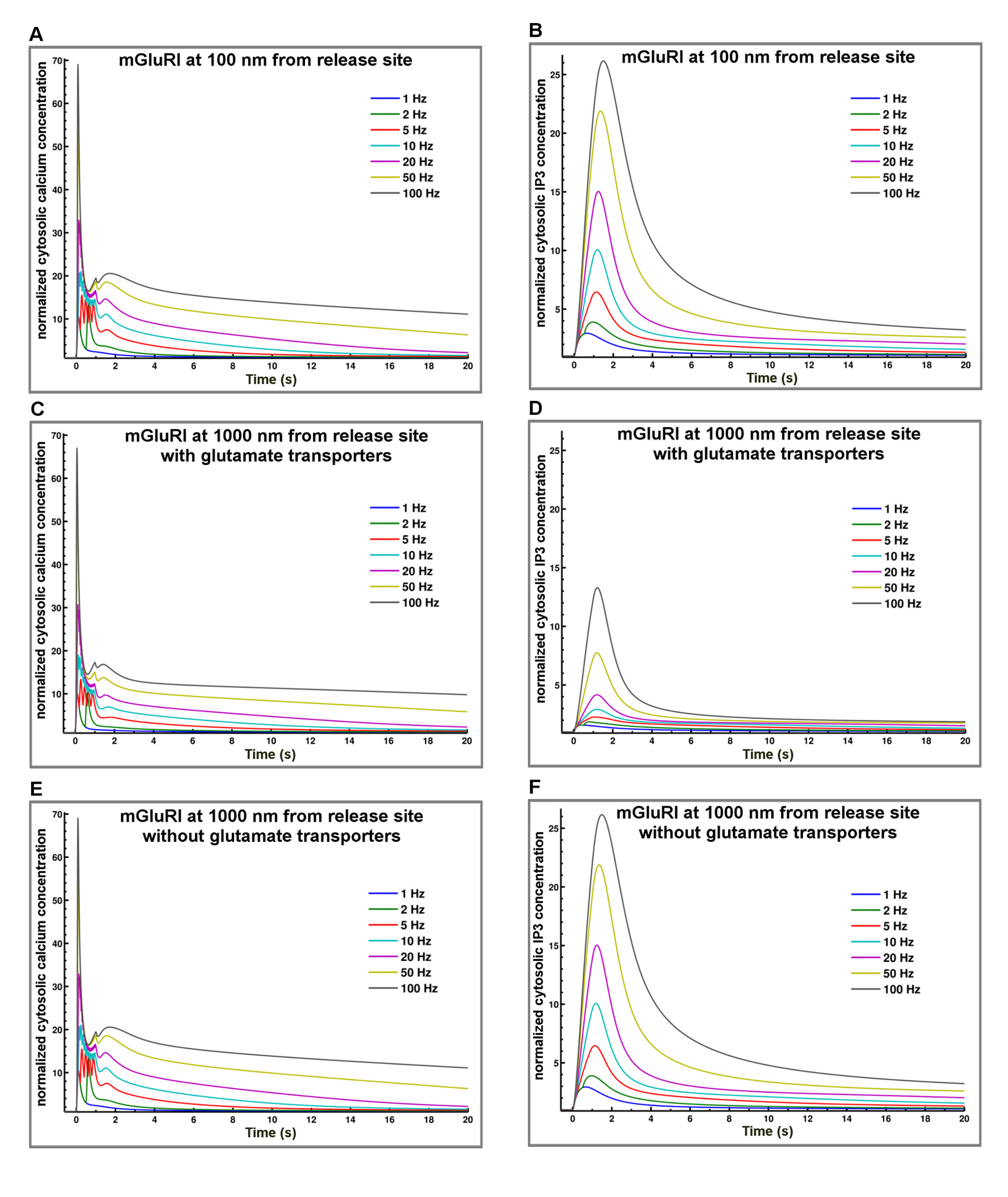

Supplement: Figure S4 — Effects of stimulation frequency, glutamate transporters, and mGluRI location on glutamate-mediated calcium and IP3 dynamics. (A, B) Temporal evolution of cytosolic calcium (A) and IP3 (B) concentrations generated by glutamate receptor (AMPA, NMDA and mGluRI receptors) activation in response to various stimulation frequencies (color lines) with mGluRI receptors located at 100 nm from the release site. (C, D) Corresponding temporal evolution of cytosolic calcium (C) and IP3 (D) concentrations generated by glutamate receptor (AMPA, NMDA and mGluRI receptors) activation in response to various stimulation frequencies (color lines) with mGluRI receptors located at 1000 nm (B) from the release site in the presence of glutamate transporters. Note the small effect of the location of mGluRI on calcium dynamics as opposed to the large effect on IP3 dynamics. (E, F) Temporal evolution of cytosolic calcium (E) and IP3 (F) concentrations generated by glutamate receptor (AMPA, NMDA and mGluRI receptors) activation in response to various stimulation frequencies (color lines) with mGluRI receptors located at 1000 nm (B) from the release site in the absence of glutamate transporters. Note that absence of glutamate transporters reversed the effects of the localization of mGluRI away from the release site for both calcium and IP3 dynamics. Calcium and IP3 responses were normalized to respective cytosolic basal concentrations (60 and 100 nM). (TIFF) [file pone.0028380.s004.tiff]

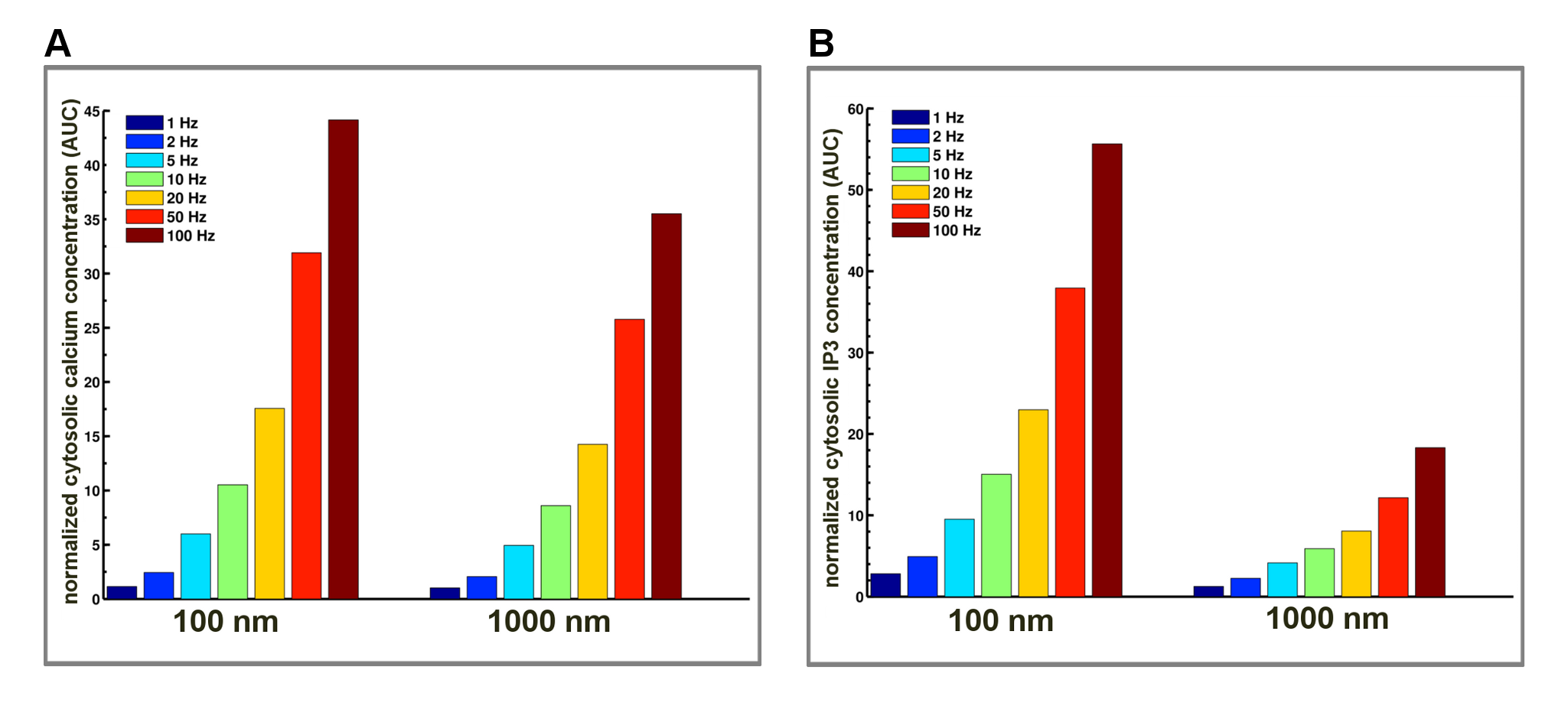

Supplement: Figure S5 — Effects of stimulation frequency and mGluRI location on the respective contribution of ionotropic and metabotropic glutamate receptors on integrated calcium and IP3 dynamics. (A, B) Histograms represent the AUC for calcium (A) and IP3 (B) dynamics generated by activation of mGluRI and ionotropic glutamate receptors (AMPA/NMDA) with mGluRI located at 100 nm or 1000 nm from the release site at various stimulation frequencies. AUC values were normalized to the AUC value calculated for AMPA/NMDA response following one release event. (TIFF) [file pone.0028380.s005.tiff]

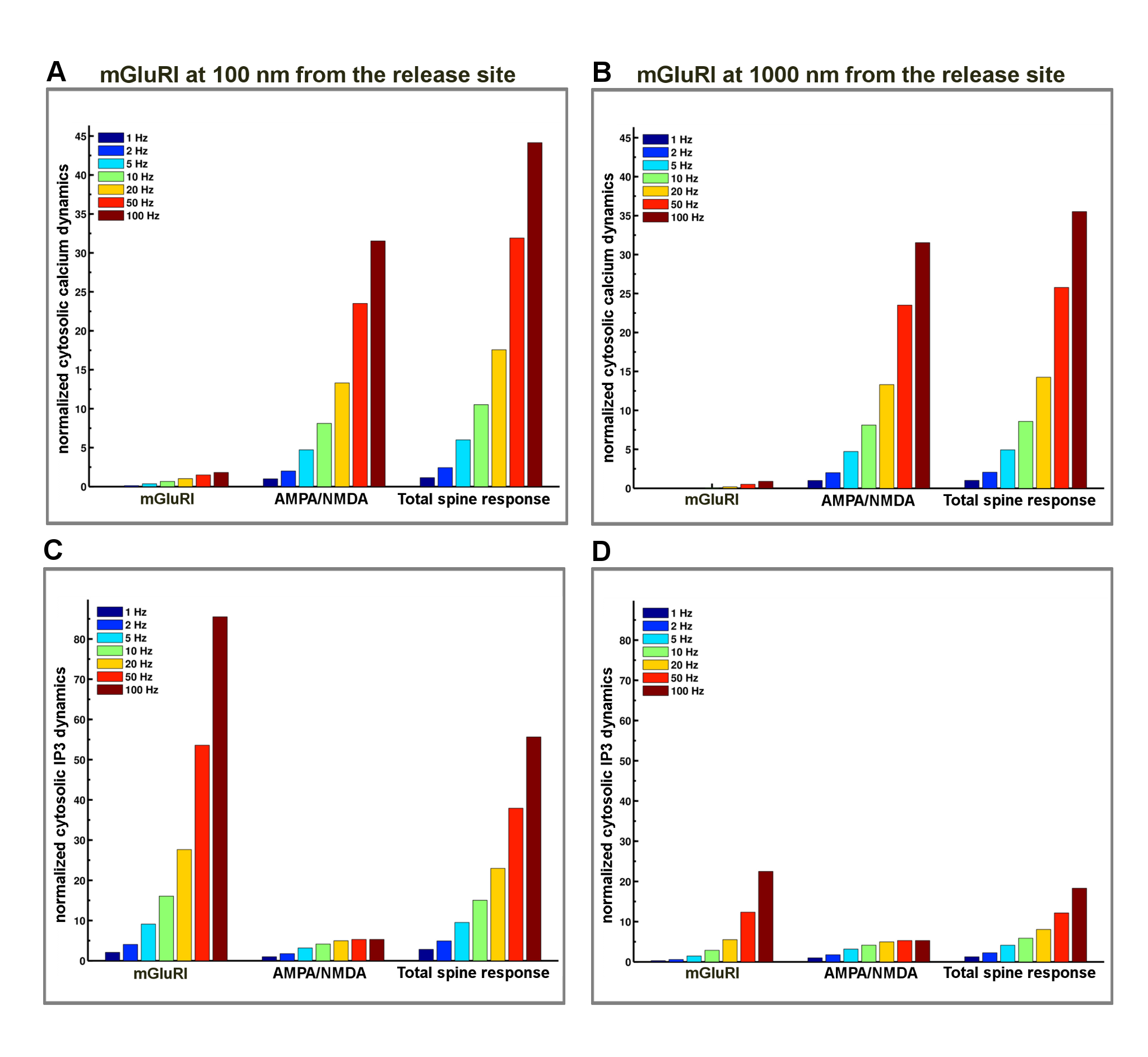

Supplement: Figure S6 — Interactions between ionotropic and metabotropic receptors in glutamate-mediated calcium and IP3 transients in dendritic spines. (A, B) Histograms represent the quantification of the AUC for calcium dynamics generated by activation of mGluRI, ionotropic glutamate receptors (AMPA/NMDA), and all receptors (total spine response) with mGluRI located at 100 nm (A) or 1000 nm (B) from the release site at various stimulation frequencies. Note the significant supra-additive effect of the combined activation of all the receptors on calcium dynamics. (C, D) Corresponding histograms of the quantification of AUC for IP3 dynamics generated by activation of mGluRI, ionotropic glutamate receptors (AMPA/NMDA), and all receptors (total spine response) with mGluRI located at 100 nm (C) or 1000 nm (D) from the release site at various stimulation frequencies. Note the inhibitory effect of the activation of AMPA/NMDA receptors on the total response to glutamate stimulation, especially at high stimulation frequency. In all cases, AUC values were normalized to the AUC value calculated for AMPA/NMDA response following one release event. (TIFF) [file pone.0028380.s006.tiff]
