# Supplementary material for: Simulation of Postsynaptic Glutamate Receptors Reveals Critical Features of Glutamatergic Transmission
Source: PLoS One. 2011 Dec 15;6(12):e28380. doi: 10.1371/journal.pone.0028380 (PMC3240618; doi:10.1371/journal.pone.0028380)
Supplement: Table S1 — Reaction equations, rate laws, and kinetic parameters of the mGluRI and calcium dynamics models. (DOC) [file pone.0028380.s007.doc]

**Supporting** Table 1. Reaction equations, rate laws, and kinetic parameters of the mGluRI and calcium dynamics models

| **Group (Figure 1B)** | **Mechanisms** | **Reactions** | **Kinetic parameters** | **References** |
| --- | --- | --- | --- | --- |
| **mGluRI PARAMETERS** | | | | |
| **Receptor parameters** Reaction#1 | Receptor activation | J0=J0_k1*R-J0_k2*Ra; J1=J1_k1*LR-J1_k2*LRa; J2=J2_k1*R_Gaq_GDP_bg-J2_k2*Ra_Gaq_GDP_bg; J3=J3_k1*LR_Gaq_GDP_bg-J3_k2*LRa_Gaq_GDP_bg | J0_k1=0.001 ms^-1^; J0_k2=1 ms^-1^; J1_k1=0.001 ms^-1^; J1_k2=1 ms^-1^; J2_k1=0.001 ms^-1^; J2_k2=1 ms^-1^; J3_k1=0.001 ms^-1^; J3_k2=1 ms^-1^ | [36] |
|  | Glutamate binding | J4=J4_k1*R*L-J4_k2*LR; J5 = J5_k1*Ra*L-J5_k2*LRa; J6=J6_k1*R_Gaq_GDP_bg*L-J6_k2*LR_Gaq_GDP_bg; J7=J7_k1*Ra_Gaq_GDP_bg*L-J7_k2*LRa_Gaq_GDP_bg | J4_k1=5000 mM^-1^.ms^-1^; J4_k2=20 ms^-1^; J5_k1=5000 mM^-1^.ms^-1^; J5_k2=20 ms^-1^; J6_k1=5000 mM^-1^.ms^-1^; J6_k2=20 ms^-1^; J7_k1=5000 mM^-1^.ms^-1^; J7_k2=20 ms^-1^ | [59,60] |
|  | G protein binding | J8=J8_k1*R*Gaq_GDP_bg-J8_k2*R_Gaq_GDP_bg; J9=J9_k1*Ra*Gaq_GDP_bg-J9_k2*Ra_Gaq_GDP_bg; J10=J10_k1*LRa*Gaq_GDP_bg-J10_k2*LRa_Gaq_GDP_bg; J11=J11_k1*LR*Gaq_GDP_bg-J11_k2*LR_Gaq_GDP_bg | J8_k1=0.6 mM^-1^.ms^-1^; J8_k2=0.001 ms^-1^; J9_k1=0.6 mM^-1^.ms^-1^; J9_k2=0.001 ms^-1^; J10_k1=0.1 mM^-1^.ms^-1^; J10_k2=6.5e-5 ms^-1^; J11_k1=0.1 mM^-1^.ms^-1^; J11_k2=6.5e-5 ms^-1^ | [36,61] |
| **Transduction parameters** Reactions#2 to 6 | GDP exchange | J12=J12_k1*Ra_Gaq_GDP_bg-J12_k2*GDP*Ra_Gaq0_bg; J15=J15_k1*LRa_Gaq_GDP_bg-J15_k2*GDP*LRa_Gaq0_bg | J12_k1=0.003 ms^-1^; J12_k2=30 mM^-1^.ms^-1^; J15_k1=0.003 ms^-1^; J15_k2=30 mM^-1^.ms^-1^ | [62,63,64,65] |
|  | GTP exchange | J13=J13_k1*Ra_Gaq0_bg*GTP-J13_k2*Ra_Gaq_GTP_bg; J16=J16_k1*LRa_Gaq0_bg*GTP-J16_k2*LRa_Gaq_GTP_bg | J13_k1=30 mM^-1^.ms^-1^; J13_k2=5e-6 ms^-1^; J16_k1=30 mM^-1^.ms^-1^; J16_k2=5e-6 ms^-1^ | [63,64,65] |
|  | G protein activation | J14=J14_k1*Ra_Gaq_GTP_bg; J17=J17_k1*LRa_Gaq_GTP_bg | J14_k1=1.5e-8 ms^-1^; J17_k1=1.5e+2 ms^-1^ | [66] |
|  | GTPase | J18=J18_k1*GaqGTP | J18_k1=0.000001 ms^-1^ | [63,65] |
|  | G protein trimerization | J19=J19_k1*GaqGDP*Gbg | J19_k1= 000 mM^-1^.ms^-1^ | [55] |
| **IP3 FORMATION** | | | | |
| **PLC**  Reaction#7 | calcium binding | J20=J20_k1*PLC_Gq_PIP2*Ca-J20_k2*PLC_Ca_Gq_PIP2 | J20_k1=100 mM^-1^.ms^-1^; J20_k2=0.03 ms^-1^ | [36,55,58] |
|  | G-protein binding | J21=J21_k1*PLC_Ca_PIP2*GaqGTP-J21_k2*PLC_Ca_Gq_PIP2; J22=J22_k1*PLC_Ca*GaqGTP-J22_k2*PLC_Ca_Gq | J21_k1=80 mM^-1^.ms^-1^; J21_k2=0.06 ms^-1^; J22_k1=80 mM^-1^.ms^-1^; J22_k2=0.06 ms^-1^ | [54,55,67] |
|  | IP3 & DAG production | J23=J23_k1*PLC_Ca_PIP2; J24=J24_k1*PLC_Ca_Gq_PIP2 | J23_k1=0.08 mM^-1^.ms^-1^; J24_k1=0.56 mM^-1^.ms^-1^ | [54,55,67,68] |
|  | PIP2 binding | J25=J25_k1*PLC*PIP2-J25_k2*PLC_Ca_PIP2; J26=J26_k1*PLC_Ca_Gq*PIP2-J26_k2*PLC_Ca_Gq_PIP2 | J25_k1=200 mM^-1^.ms^-1^; J25_k2=0.26 ms^-1^ ; J26_k1=200 mM^-1^.ms^-1^; J26_k2=0.26 ms^-1^ | [54,55,69] |

**Supporting Table 1. Reaction equations, rate laws, and kinetic parameters of the mGluRI and calcium dynamics models (continued)**

| **IP3 METABOLISM** | | | | |
| --- | --- | --- | --- | --- |
| **IP3 5-phosphatase** Reaction#8 | IP3 binding | J27=J27_k1*IP5P*IP3-J27_k2*IP5P_IP3 | J27_k1=59 mM^-1^.ms^-1^; J27_k2=0.072 ms^-1^ | [37] |
|  | IP2 formation | J28=J28_k1*IP5P_IP3 | J28_k1=0.018 ms^-1^ | [55] |
| **IP3 3-kinase** Reaction#9 | calcium binding | J29=J29_k1*IP3K*2Ca-J29_k2*IP3K_Ca | J29_k1=111 mM^-1^.ms^-1^; J29_k2=0.1 ms^-1^ | [37] |
|  | IP3 binding | J30=J30_k1*IP3K_2Ca*IP3-J30_k2*IP3K_2Ca_IP3 | J30_k1=500 mM^-1^.ms^-1^; J30_k2=0.08 ms^-1^ | [37] |
|  | IP4 formation | J31=J31_k1*IP3K_2Ca_IP3 | J31_k1=0.02 ms^-1^ | [55] |
| **IP3 receptor** Reactions#10 & 11 | calcium binding | J32=J32_k1*IP3R*4Ca-J32_k2*IP3R_4Ca | J32_k1=81.9 mM^-1^.ms^-1^; J32_k2=0.005 ms^-1^ | [52,53,55,70,71] |
|  | IP3 binding | J33=J33_k1*IP3R*IP3-J33_k2*IP3R_IP3 | J33_k1=500 mM^-1^.ms^-1^; J33_k2=250.8 ms^-1^ | [52,53,55,70,71] |
|  | IP3R calcium channel | J34=J34_k1*IP3R_IP3*Ca-J34_k2*IP3R_Open | J34_k1=800 mM^-1^.ms^-1^; J34_k2=0.2 ms^-1^ | [52,53,55,70,71] |
| **CALCIUM DYNAMICS** | | | | |
| **SERCA** Reaction#12 | calcium binding | J35=J35_k1*SERCA*2Ca-J35_k2*SERCA_2Ca | J35_k1=45 mM^-1^.ms^-1^; J35_k2=0.032 ms^-1^ | [54,55,72,73] |
|  | calcium uptake | J36=J36_k1*SERCA_2Ca | J36_k1=0.15 ms^-1^ | [54,55,72,73] |
| **Calcium/sodium exchanger** Reaction#14 | calcium binding | J37=J37_k1*NCX*2Ca-J37_k2*NCX_2Ca | J37_k1=940 mM^-1^.ms^-1^; J37_k2=4 ms^-1^ | [55] |
|  | calcium uptake | J38=J38_k1*NCX_2Ca | J38_k1=80 ms^-1^ | [55] |
| **PMCA** Reaction#15 | calcium binding | J39=J39_k1*PMCA*Ca-J39_k2*PMCA_Ca | J39_k1=250 mM^-1^.ms-1; J39_k2=0.2 ms^-1^ | [54,55,74] |
|  | calcium uptake | J40=J40_k1*PMCA_Ca | J40_k1=0.05 ms^-1^ | [54,55,74] |
| **Leak channels** Reactions#13 & 16 | calcium leak from cleft | J41=J41_k1*Ca_Cleft | J41_k1=0.001 ms^-1^ | [55] |
|  | calcium leak from ER | J42=J42_k1*CaER | J42_k1=0.0015 ms^-1^ | [55] |
